# Lung Ultrasound to Phenotype Chronic Lung Allograft Dysfunction in Lung Transplant Recipients. A Prospective Observational Study

**DOI:** 10.3390/jcm10051078

**Published:** 2021-03-05

**Authors:** Jesper Rømhild Davidsen, Christian B. Laursen, Mikkel Højlund, Thomas Kromann Lund, Klaus Nielsen Jeschke, Martin Iversen, Anna Kalhauge, Elisabeth Bendstrup, Jørn Carlsen, Michael Perch, Daniel Pilsgaard Henriksen, Hans Henrik Lawaetz Schultz

**Affiliations:** 1 South Danish Center for Interstitial Lung Diseases (SCILS), Odense University Hospital, 5000 Odense, Denmark; christian.b.laursen@rsyd.dk; 2 Department of Respiratory Medicine, Odense University Hospital, 5000 Odense, Denmark; 3 Odense Respiratory Research Unit (ODIN), Department of Clinical Research, University of Southern Denmark, 5000 Odense, Denmark; 4 Odense Patient Data Explorative Network, Odense University Hospital, 5000 Odense, Denmark; 5 Department of Clinical Biochemistry and Pharmacology, Odense University Hospital, 5000 Odense, Denmarkdph@rsyd.dk (D.P.H.); 6 Department of Cardiology, Section for Lung Transplantation, Copenhagen University Hospital, Rigshospitalet, 2100 Copenhagen, Denmark; thomas.kromann.lund@regionh.dk (T.K.L.); martin.iversen@regionh.dk (M.I.); joern.carlsen@regionh.dk (J.C.); michael.perch@regionh.dk (M.P.); hans.henrik.lawaetz.schultz.01@regionh.dk (H.H.L.S.); 7 Department of Respiratory Medicine, Copenhagen University Hospital, Hvidovre Hospital, 2650 Hvidovre, Denmark; klaus.nielsen.jeschke@regionh.dk; 8 Department of Radiology, Copenhagen University Hospital, Rigshospitalet, 2100 Copenhagen, Denmark; anna.kalhauge@regionh.dk; 9 Center for Rare Lung Diseases, Department Respiratory Diseases and Allergy, Aarhus University Hospital, 8200 Aarhus, Denmark; karbends@rm.dk; 10 Department of Clinical Medicine, Faculty of Health and Medical Sciences, University of Copenhagen, 2200 Copenhagen, Denmark

**Keywords:** bronchiolitis obliterans syndrome, chronic lung allograft dysfunction, lung transplantation, lung ultrasound, pleuroparenchymal fibroelastosis, restrictive allograft syndrome

## Abstract

Background: Bronchiolitis obliterans syndrome (BOS) and restrictive allograft syndrome (RAS) are two distinct phenotypes of chronic lung allograft dysfunction (CLAD) in lung transplant (LTx) recipients. Contrary to BOS, RAS can radiologically present with a pleuroparenchymal fibroelastosis (PPFE) pattern. This study investigates lung ultrasound (LUS) to identify potential surrogate markers of PPFE in order to distinguish CLAD phenotype RAS from BOS. Methods: A prospective cohort study performed at a National Lung Transplantation Center during June 2016 to December 2017. Patients were examined with LUS and high-resolution computed tomography of the thorax (HRCT). Results: Twenty-five CLAD patients (72% males, median age of 54 years) were included, corresponding to 19/6 BOS/RAS patients. LUS-identified pleural thickening was more pronounced in RAS vs. BOS patients (5.6 vs. 2.9 mm) compatible with PPFE on HRCT. LUS-identified pleural thickening as an indicator of PPFE in RAS patients’ upper lobes showed a sensitivity of 100% (95% CI; 54–100%), specificity of 100% (95% CI; 82–100%), PPV of 100% (95% CI; 54–100%), and NPV of 100% (95% CI; 82–100%). Conclusion: Apical pleural thickening detected by LUS and compatible with PPFE on HRCT separates RAS from BOS in patients with CLAD. We propose LUS as a supplementary tool for initial CLAD phenotyping.

## 1. Introduction

Chronic lung allograft dysfunction (CLAD) is the leading cause of morbidity and mortality in adult lung transplant recipients, affecting more than 50% surviving beyond five years after lung transplantation (LTx) [1]. Numerous risk factors are associated with CLAD development, such as primary graft dysfunction (PGD) [2], acute cellular rejection (ACR) [3], gastroesophageal reflux disease (GERD), donor smoking history [4], infections, and antibody-mediated rejection (AMR) [5,6]. Identification of CLAD development is accomplished by frequent monitoring of lung physiological parameters including forced expiratory volume in one second (FEV1), forced vital capacity (FVC), and total lung capacity (TLC) during surveillance follow-up [7]. Previously CLAD was synonymous with bronchiolitis obliterans syndrome (BOS) defined as a persistent decline in FEV1 [5]. However, in 2011 a novel CLAD phenotype-restrictive allograft dysfunction (RAS)-was introduced and characterized by a combined decline in FEV1 and TLC with concurrent presence of pleuroparenchymal fibrotic changes on a high-resolution computed tomography (HRCT) [8,9,10,11]. Ofek et al. has described the presence of pleuroparenchymal fibroelastosis (PPFE) as the histopathological correlate of RAS in autopsy and biopsy material [12]. For RAS, the radiological criteria were updated in 2019 to include all presence of parenchymal, with or without pleural-based, persistent opacities [13,14]. In contrast to BOS no documented treatment exists for RAS [15]. Furthermore, RAS differs from BOS by a decreased survival at primary- and re-LTx [10,11,16].

There is only limited experience with lung ultrasound (LUS) as part of LTx follow-up. LUS has primarily been used in the early post-operative phase after LTx in order to diagnose and monitor acute complications such as pleural effusion, pneumonia, and atelectasis [17,18,19,20]. In interstitial lung disease (ILD), lung parenchyma and pleura possess an increased tissue density, which can be demonstrated by LUS findings as a significant number of B-line reverberation artifacts as part of the interstitial syndrome (IS), and pleural thickening [21,22,23,24,25,26,27].

Due to LUS’ diagnostic properties within ILD, LUS might be a novel and supplementary diagnostic tool for CLAD phenotyping [5]. No solid evidence exists on this topic. Yet, one case report has focused on LUS as a potential tool to diagnose RAS based on the high diagnostic accuracy of LUS to identify interstitial and pleural pathology in patients with ILD including PPFE [28]. However, prospective studies comparing LUS with HRCT findings as gold standard in a series of cases with CLAD are warranted to generate more information about the role of LUS in this context. This study aimed to investigate whether LUS is usable to identify potential surrogate markers of a radiological PPFE pattern in order to distinguish CLAD phenotype RAS from BOS.

## 2. Materials and Methods

### 2.1. Setting

In Denmark LTx is only performed at the National Lung Transplantation Centre in Copenhagen, where clinical follow-up is also performed the first two years post-LTx. Hereafter, clinical follow-up of lung transplant recipients can also be managed at tertiary specialist centers at the Departments of Respiratory Medicine at Aarhus and Odense University Hospital using the same follow-up protocol [29,30]. The inclusion took place at all these three centers.

### 2.2. Study Design

A prospective triple center study based on individual cross-sectional clinical evaluation of lung transplant recipients diagnosed with CLAD with inclusion from 16 June 2016 through 7 December 2017.

### 2.3. Study Cohort and CLAD Definition

Eligible double LTx (DLTx) patients with a newly diagnosis of CLAD verified by a bodyplethysmography and HRCT were recruited. Phenotyping CLAD was based on the following definitions [13,14]:

- BOS: A persistent decline in FEV1 of ≥20% compared to the average of the two best obtained post-LTx baseline values, where other causes of lung function decline were ruled out (e.g., infection, and pleural effusion).
- RAS: As stated above for BOS but with an additional decline in TLC of ≥10% compared to the best post-LTx baseline values and combined with persistent radiographic abnormalities including the presence of parenchymal with (PPFE pattern) or without pleural-based opacities.

According to Sato et al. CLAD was estimated to appear with a distribution of BOS:RAS corresponding to 4:1 [8]. As a proof-of-concept approach, and in order to obtain representative LUS data for both CLAD phenotypes, we planned to include 25 patients in total aiming for at least five patients with RAS.

### 2.4. Study Plan

In relation to a new-onset diagnosis of CLAD, LUS was performed within a time window of maximum three months before or after HRCT in order to accomplish a real time cross-sectional assessment of potential LUS findings as surrogate markers of HRCT pleuroparenchymal pathology (e.g., surrogate findings on LUS compatible with lung fibrosis as part of a radiological PPFE pattern).

### 2.5. LUS

#### 2.5.1. Scanning Protocol and Equipment

**Figure 1:**
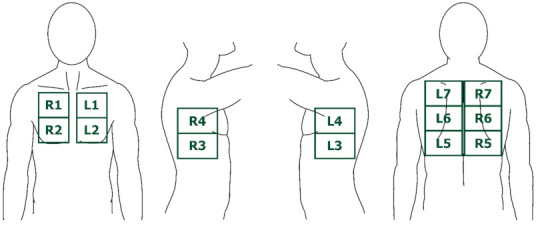
The seven lung ultrasound (LUS) zones on each hemithorax corresponding to the anterior, lateral, and posterior thorax wall. Abbreviations: L = left. R = right.

Consistent with previous studies by this research group the lung transplant recipients were examined in a sitting position, and thorax was systematically scanned in fourteen different zones (seven zones on each hemithorax (i.e., zones R (right) 1–7 and L (left) 1–7) covering the anterior, lateral and posterior thorax wall with one LUS record stored per zone (Figure 1) [19,31]. LUS was performed by three experts (J.R.D., H.H.L.S., K.N.J.) using a GE Logiq S8 (GE Healthcare, Milwaukee, WI, USA) ultrasound system with a linear ML6-15 (6–15 MHz) and abdominal C1-5 (1–5 MHz) transducer with a focus setting of 1.5–2 cm and a depth of 4 and 15 cm, respectively.

#### 2.5.2. Selected Outcome Variables

**Figure 2:**
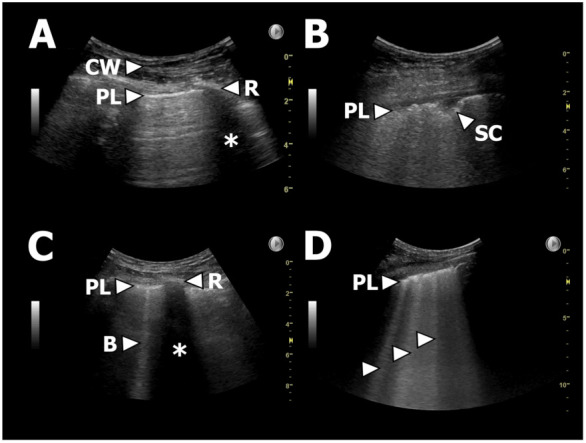
Examples of LUS findings. (**A**): Normal LUS. PL = pleural line, CW = chest wall, R = rib, * = rib shadowing. (**B**): Thickened/fragmented pleura and a small subpleural consolidation. PL = pleural line, SC = subpleural consolidation. (**C**): Single B-line. PL = pleural line, R = rib, * = rib shadowing, B = B-line. (**D**): Multiple B-lines as part of, e.g., IS. PL = pleural line, arrows = B-lines.

B-lines, IS, and pleural thickening are LUS findings known to be associated with presence of fibrosis identified on HRCT [21,22,23,24,25,26,27]. We hypothesized that presence of these LUS findings in new-onset CLAD could act as surrogate markers of a radiological PPFE pattern verified on HRCT in patients with RAS. In such LUS could be a supplementary diagnostic tool to phenotype RAS from BOS in whom these LUS findings are not expected [32]. We used the following definitions (Figure 2):

*Number of B-lines:* B-lines were defined as vertical reverberation artefacts originating from the pleural line extending uninterrupted to the edge of the screen on the ultrasound machine without fading (previously termed “comet-tails”) [21].

*Interstitial syndrome (IS):* ≥3 B-lines in ≥2 anterior or lateral zones on each hemithorax [22,23].

*Upper lobe IS*: ≥ 3 B-lines in in both zone R/L1 and R/L7.

*Pleural thickening:* Pleura thickness >1 mm regardless a normal or abnormal irregular or fragmented presence of pleura [26,33].

One LUS expert (CBL) independently and blinded reviewed the LUS records and confirmed the findings obtained by the recruiting physicians (JRD, HHLS, KNJ).

### 2.6. HRCT

#### 2.6.1. Scanning Protocol

All patients had a HRCT scan performed in a supine position from apex to below the costophrenic angle in 64 (or more) slice scanners, and a standard lung algorithm was used to obtain 1 mm non-overlapping slices that were reviewed in a lung window setting (window width 1500 Hounsfield units (HU); window level−500 HU) [19].

#### 2.6.2. Selected Outcome Variables

All HRCT scans were evaluated corresponding to pathological findings in central and peripheral areas of the right upper, middle, and lower lobes, and the left upper, and lower lobes corresponding to 10 areas in total [34,35]. The HRCT scans were evaluated by two pulmonologists and one radiologist experienced in HRCT (T.K.L, M.I., A.K.) who reached consensus according to dichotomised presence of specific radiological findings compatible with fibrotic interstitial or pleural affection of the lung allografts. According to Jacob et al. we categorised parenchymal fibrosis as present if either criterion 1, 2 and 3, criteria 1 and 3, or 2 and 3 as described below were fulfilled [36,37]:

1. Honeycombing
2. Tractions bronchiectasis
3. Volume loss

Supportive fibrotic parenchymal findings to the above criteria were ≥1 of following: Reticulation, ground glass opacity (GGO), septal thickening (i.e., thickening of the intra- and/or interlobular septa). Fibrotic pleural affection was stated when pleural thickening was observed.

### 2.7. Statistical Analysis

The main outcomes were proportions of specific LUS and HRCT findings according to the selected outcome variables. Continuous variables as baseline demographic data were expressed as median(s) with corresponding interquartile range (IQR), and descriptive categorical data were expressed as numbers and proportions. To evaluate the diagnostic accuracy for specific LUS findings indicating PPFE in RAS (reference test), we calculated and expressed sensitivity, specificity, positive predictive values (PPV), and negative predictive values (NPV) and their 95% confidence intervals (CIs). The Wilcoxon rank-sum or Chi2 test were used for comparison of outcomes expressed as proportion with a two-sided significance of 5%. All analyses were performed using Stata SE 16.1 (StataCorp, College Station, TX, USA).

## 3. Results

### 3.1. Patient Characteristics

Twenty-five patients with CLAD were included corresponding to BOS:RAS of 19:6 with an overall male predominance of 72.0% and a median age of 54 years (IQR 37 to 57 years). Emphysema in an equal distribution with and without alpha-1 antitrypsin deficiency (A1AD) constituted the main underlying disease category for LTx (48%) followed by cystic fibrosis (24%), and different ILD subtypes (24%). In BOS and RAS patients, the lung physiology was represented by an average decline from best obtained post-LTx FEV1 and TLC of 49% and 38%, respectively. Other baseline characteristics are presented in Table 1. The median time-window between completion of LUS and HRCT was 10 days (IQR 2 to 43 days).

### 3.2. LUS Findings

**Figure 3:**
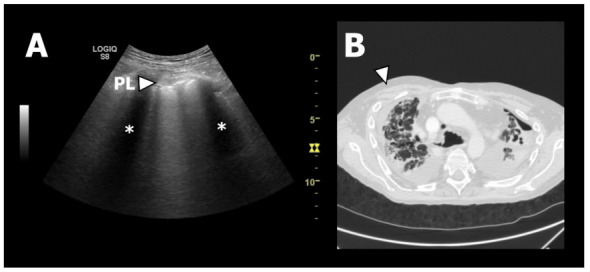
LUS and HRCT findings from a patient with RAS. (**A**): Lung ultrasound image from zone R1. At the top of the chest wall structures can be seen as a grey area. Just below the chest wall the pleural line (PL) can be seen as a horizontal white line. It has an abnormal appearance since it is thickened and fragmented. The two black areas (*) is caused by “shadowing” due to the ultrasound waves being absorbed and reflected by the ribs. (**B**): Axial HRCT image of the upper lobes showing fibrotic pleural and septal thickening consistent with PPFE. The white arrow corresponds to LUS zone R1 presented in A.

In total 350 LUS records (25 patients with each 14 LUS zones) were reviewed and interpreted with findings as presented in Table 2. A significant majority of patients with RAS presented with pleural thickening, when compared to patients with BOS (Figure 3). In RAS patients, the pleural line was solely characterised as being fragmented, and in addition significantly thicker than the pleural line observed in BOS patients corresponding to 5.6 mm and 2.9 mm, respectively (Supplementary Table S1). Additionally, the proportion of patients with ≥3 B-lines was more predominant among RAS patients than in BOS patients. No consolidations were identified in either group.

### 3.3. HRCT Findings

The detailed interpretation of 250 HRCT areas (25 patients with each 10 HRCT areas) formed the basis for the primary observation that a higher proportion of RAS than BOS patients presented with findings as GGO, consolidation, septal and pleural thickening, reticulation, traction bronchiectasis, and volume loss. All these radiological findings indicate parenchymal affection with manifest fibrosis consistent with 83.3–100.0% of the RAS patients being classified as having fibrosis, but notable none with presence of honeycombing (Table 3). When restricting to presence of parenchymal fibrosis and pleural thickening to the apical parts of the lungs (i.e., right and left upper central and peripheral areas), we found a statistically significant higher proportions of these findings in patients with RAS compared to BOS, a signal that was not found for GGO (Supplementary Table S2).

### 3.4. Diagnostic Accuracy of LUS

IS as a LUS indicator of PPFE in patients with RAS had a sensitivity of 17% (95% CI; 0–64%), specificity of 100% (95% CI; 82–100%), PPV of 100% (95% CI; 3–100%), and NPV of 79% (95% CI; 58–93%). The upper lobe IS definition had a sensitivity of 0% (95% CI; 0–46%), specificity of 100% (95% CI; 82–100%), PPV not applicable, and NPV of 76% (95% CI; 55–91%) (Table 4).

When focusing on pleural thickening in either anterior or posterior apical zones (i.e., zones R/L1 and R/L7) as a PPFE indicator, we found sensitivities of 67–83% (95% CI; 22–100%), a specificity of 100% (95% CI; 82–100%), PPV of 100% (95% CI; 40–100%), and NPV of 90–95% (95% CI; 64–99%) (Table 4). When analysing for a composite indicator of either pleural thickening in zones R/L1 or zones R/L7, the sensitivity was 100% (95% CI; 54–100%), specificity of 100% (95% CI; 82–100%), PPV of 100% (95% CI; 54–100%), and NPV of 100% (95% CI; 82–100%) (Table 4).

## 4. Discussion

This is the first observational study using a prospective approach to investigate LUS in a post-LTx setting as a novel and potential diagnostic tool to phenotype CLAD subtypes RAS from BOS. We have demonstrated that the proportion of patients with findings consistent with pleural thickening in the lung apices using LUS was significantly higher among RAS than BOS patients. Furthermore, that LUS had a high diagnostic accuracy to identify pleural thickening in RAS patients as a surrogate measure of a radiological PPFE pattern. In addition, the pleural thickness measured using LUS was almost doubled in size in RAS compared to BOS patients.

### 4.1. LUS Findings

We hypothesized that a PPFE pattern on HRCT in RAS patients would exhibit LUS findings representing parenchymal and pleural affection as already recognized in other fibrotic ILDs in case of IS and pleural thickening [38,39]. Additionally, such LUS findings would not appear in patients with BOS compatible with other obstructive conditions such as COPD and emphysema [32].

Current experience with the use of LUS in LTx settings is sparse and has mainly focused on identifying complications in the early post-LTx course [19,20]. To our knowledge, no previous studies have investigated the use of LUS in order to distinguish CLAD phenotypes. Our group has previously reported the association between HRCT proven PPFE and LUS findings suggestive of PPFE in one RAS patient [28]. In this case IS was detected by LUS using the international consensus definition of IS, but in the present study only one patient fulfilled the IS criteria despite the proportion of RAS patients in general had more B-lines compared to BOS patients [22,23] (Table 2). This is also supported by the low sensitivity of IS and upper lobe IS of 17% and 0%, respectively, and thus IS seems to be an inappropriate surrogate marker of RAS. In six RAS patients, LUS identified fragmented pleural thickening, a well-known feature observed in other fibrotic ILDs representing distortion and “pleural traction” from subpleural parenchymal fibrosis [21,25]. In our case series these changes where due to a radiological PPFE pattern. We found a high diagnostic accuracy of LUS to detect surrogate markers of a PPFE pattern in RAS patients in terms of pleural thickening in apical LUS zones (Table 4). This new “RAS-sign” might be expected, nevertheless, this is novel and clinically important knowledge has not previously been described in CLAD phenotyping. Hence, our results indicate that LUS identified pleural thickening confirmed by the RAS-sign in patients at suspicion of developing CLAD may increase the likelihood of RAS rather than BOS.

### 4.2. HRCT Findings

Normally idiopathic PPFE is radiologically characterised by predominantly apical pleural and subpleural fibrosis, subpleural reticulation, traction bronchiectasis, and possibly GGO, which may be the most plausible explanation that honeycombing (as a fibrotic feature) was not observed in any of the RAS patients [40]. Not surprisingly, apical fibrosis was present in all RAS patients compatible with a verified PPFE pattern on HRCT whereas it was only observed in 21% of the BOS patients (*p* = 0.00058). In BOS patients, findings such as air trapping, bronchiectasis, and peribronchial thickening indicating airway pathology dominated as expected. The high proportion of apical fibrosis in our RAS patients differ from observations by Horie M et al., where apical interstitial findings were present in only 48% of their RAS patients compared to 18% in BOS patients [41]. The fact that Horie M. et al. conducted low dose CT scans at regular intervals (i.e., 3, 6, 9, 12, 18, 24 months post-LTx) may explain the difference in presence of RAS findings as they potentially identified findings suggestive for RAS earlier compared to our approach, where HRCT was performed on clinical and lung physiological suspicion of CLAD.

Though pleural thickening on HRCT appeared in up to one third of the BOS patients, this feature was present in all RAS patients corresponding to the upper, central, and lower peripheral lung areas, and in agreement with the above-mentioned LUS findings.

### 4.3. Strengths and Limitations

A strength of this study was the nationwide approach with consistent inclusion from all three LTx referral centers, thus covering an entire nation’s CLAD patients diagnosed during the observation period. Thus, all patients had CLAD consensus confirmed on a multidisciplinary conference on basis of lung physiology and HRCT independently evaluated by at least two experienced specialists.

LTx is a treatment option accessible to only highly selected patients with chronic end-stage lung diseases [42]. In such, the apparent main limitation of this study was the small study size. Our observations may therefore not necessarily correspond to findings from other CLAD cohorts. However, when focusing on the study size it should be taken into the consideration that CLAD must be regarded as a rare condition in a country with a relatively low source population (population of 5.7 million in 2017). The median time between performance of HRCT (reference test) and LUS was 10 days. From a clinical perspective, this time difference is considered acceptable since CLAD development is characterized by chronic and non-acute progression with pleuroparenchymal abnormalities. However, in theory this time-gap might blur reversible findings presented on HRCT that may not appear on the following LUS or vice versa. In addition, progressive and irreversible HRCT findings may even appear worse at a later conducted LUS. Regardless of this potential LUS over-diagnostics, it would, nonetheless, have led to an additional focus on CLAD. Furthermore, attempts to assess the agreement between LUS- and HRCT findings indicating, e.g., pleural thickening was challenged by the low number of RAS patients, and the fact that positive findings in LUS zones did not consistently overlap with investigated HRCT areas, and thus did not give meaningful calculations (data not shown). We did not perform analyses for intra- or inter-observer concordance according to LUS detection of pleural thickening. However, one previous study on 38 ICU patients have shown an acceptable variance of only 0.02 cm (*p* < 0.001) among five operators when using LUS to monitor pleural length in a transversal approach [43]. Though, these data cannot necessarily be extrapolated to patients with CLAD, we to a high degree believe that the intra- and inter-observer concordance in this study is acceptable.

### 4.4. Future Research

In perspective, in CLAD patients LUS of the apical lung zones excels as a minimal time-consuming and radiation-free diagnostic tool with high diagnostic accuracy to identify surrogate findings compatible with a radiological PPFE pattern as indicative of RAS in presence of this novel RAS-sign [44]. Despite this, it is important to emphasize that LUS findings are not enough to reach a clinical diagnosis of RAS alone, but requires a composite of HRCT and lung physiology. The LUS findings in this study were obtained using patients with CLAD already confirmed by CT images and lung physiology data. It is a future theme whether LUS solely can identify pleural thickening and subpleural fibrosis without the help of clinical symptoms and radiological images in the follow-up of LTx recipients, who have no pleuropulmonary complications initially. Though, as RAS and BOS have widely different prognoses and treatment strategies, LUS could be an important future diagnostic modality in the initial CLAD investigation, and especially if suspicion of RAS. In such, it could be speculated that early LUS findings suggestive of RAS may contribute to a reduced time for CLAD phenotyping (e.g., leading to a faster ordering of HRCT and bodypletysmography). Future studies aiming to detect diagnostic accuracy of PPFE characteristics compatible with RAS should comprise time related LUS and HRCT performance on larger cohorts. Finally, but not least, as part of CLAD investigation, LUS also excels itself with the ability to exclude other causes of lung function decline (e.g., pleural effusion, pneumothorax or pneumonia) (Supplementary Table S3).

## 5. Conclusions

In patients with CLAD, apical pleural thickening separates RAS from BOS that can be detected by LUS in presence of this novel RAS-sign. In such cases, LUS can be a supplementary tool to phenotype CLAD, which may have beneficial clinical impact with shortened and timely identification of patients with RAS.

## Figures and Tables

**Table 1:** CLAD-Patient Characteristics.

Characteristic	BOS	RAS	All CLAD
	(*n* = 19)	(*n* = 6)	(*n* = 25)
Age, median (IQR)	54 (37 to 57)	51 (33 to 58)	54 (37 to 57)
Female	6 (32%)	1 (17%)	7 (28%)
Years post-LTx to CLAD (IQR)	2.6 (1.8 to 7.2)	4.9 (3.1 to 7.6)	3.1 (2.1 to 7.2)
*Underlying lung disease*			
A1AD emphysema	4 (21%)	2 (33%)	6 (24%)
COPD emphysema	5 (26%)	1 (17%)	6 (24%)
IPF	1 (5%)	0	1 (4%)
NSIP	3 (16%)	0	3 (12%)
Sarcoidosis	0	1 (17%)	1 (4%)
PAH	0	1 (17%)	1 (4%)
Anti-syntethase syndrome	1 (5%)	0	1 (4%)
CF	5 (26%)	1 (17%)	6 (24%)
re-LT	0	0	0
*Pre-LTX smoking status*			
Never smoker	9 (47%)	3 (50%)	12 (48%)
Previous smoker	10 (53%)	3 (50%)	13 (52%)
*Co-morbities*			
GERD	6 (32%)	2 (33%)	8 (32%)
*Immunosuppressive medication*			
Corticosteroids	18 (95%)	6 (100%)	24 (96%)
AZA	8 (42%)	4 (67%)	12 (48%)
MMF	5 (26%)	2 (33%)	7 (28%)
Ciclosporine	14 (74%)	3 (50%)	17 (68%)
Tacrolimus	5 (26%)	3 (50%)	8 (32%)
Everolimus	4 (21%)	0	4 (16%)
*CLAD staging*			
CLAD 0 (FEV1 > 80%)	0	0	0
CLAD 1 (FEV1 > 65–80%)	1 (5%)	1 (17%)	2 (8%)
CLAD 2 (FEV1 > 50–65%)	3 (16%)	1 (17%)	4 (16%)
CLAD 3 (FEV1 > 35–50%)	8 (42%)	2 (33%)	10 (40%)
CLAD 4 (FEV1 < 35%)	7 (37%)	2 (33%)	9 (36%)
*Change from best post-LTX, median (IQR)*			
FEV1, L	−1.77 (−1.96 to −1.20)	−1.76 (−2.15 to −1.36)	−1.77 (−1.99 to −1.34)
FEV1, percent	−49 (−68 to −38)	−48 (−48 to −35)	−48 (−60 to −35)
FVC, L	−0.95 (−1.37 to −0.43)	−2.08 (−2.31 to −1.69)	−1.12 (−1.87 to −0.52)
FVC, percent	−22 (−36 to −16)	−45 (−50 to −34)	−24 (−45 to −19)
TLC, L	0.02 (−0.30 to 0.69)	−2.45 (−3.20 to −2.00)	−0.07 (−1.33 to 0.30)
TLC, percent	0 (−4 to 7)	−38 (−53 to −32)	−1 (−15 to 6)
*Bronchial stenosis*	0	0	0

Abbreviations: A1AD = alpha-1 antitrypsin deficiency; AZA = azathioprine; CF = cystic fibrosis; CLAD = chronic lung allograft dysfunction; COPD = chronic obstructive pulmonary disease; FEV1 = forced expiratory volume in one second; FVC = forced vital capacity; GERD = gastroesophageal reflux disease; IPF = idiopathic pulmonary fibrosis; IQR = interquartile range; L = Liters; LTx = lung transplantation; MMF = Mycophenolate mofetil; NSIP = non-specific interstitial pneumonia; PAH = pulmonary arterial hypertension; TLC = total lung capacity.

**Table 2:** Specific LUS findings according to LUS scanning zones and stratified corresponding to all, BOS, and RAS patients.

	L1	L2	L3	L4	L5	L6	L7	R1	R2	R3	R4	R5	R6	R7
	No (%)	No (%)	No (%)	No (%)	No (%)	No (%)	No (%)	No (%)	No (%)	No (%)	No (%)	No (%)	No (%)	No (%)
*Pleural thickening*														
All	4 (16)	3 (12)	6 (24)	5 (20)	8 (32)	9 (36)	6 (24)	7 (28)	7 (28)	7 (28)	6 (24)	9 (36)	7 (28)	7 (28)
BOS	0 (0)	1 (5)	2 (11)	2 (11)	4 (21)	3 (16)	0 (0)	2 (11)	1 (5)	3 (16)	1 (5)	5 (26)	2 (11)	2 (11)
RAS	4 (67)	2 (33)	4 (67)	3 (50)	4 (67)	6 (100)	6 (100)	5 (83)	6 (100)	4 (67)	5 (83)	4 (67)	5 (83)	5 (83)
*Lung sliding*														
All	24 (96)	20 (80)	22 (88)	25 (100)	24 (96)	22 (88)	21 (84)	24 (96)	23 (92)	24 (96)	23 (92)	24 (96)	23 (92)	17 (68)
BOS	19 (100)	17 (89)	17 (89)	19 (100)	18 (95)	17 (89)	17 (89)	19 (100)	17 (89)	18 (95)	18 (95)	18 (95)	18 (95)	15 (79)
RAS	5 (83)	3 (50)	5 (83)	6 (100)	6 (100)	5 (83)	4 (67)	5 (83)	6 (100)	6 (100)	5 (83)	6 (100)	5 (83)	2 (33)
*>=3 B-lines*														
All	2 (8)	3 (12)	1 (4)	0 (0)	3 (12)	1 (4)	1 (4)	1 (4)	2 (8)	2 (8)	1 (4)	1 (4)	1 (4)	0 (0)
BOS	0 (0)	1 (5)	1 (5)	0 (0)	1 (5)	1 (5)	0 (0)	0 (0)	0 (0)	2 (11)	0 (0)	1 (5)	0 (0)	0 (0)
RAS	2 (33)	2 (33)	0 (0)	0 (0)	2 (33)	0 (0)	1 (17)	1 (17)	2 (33)	0 (0)	1 (17)	0 (0)	1 (17)	0 (0)
*Consolidation*														
All	0 (0)	0 (0)	0 (0)	0 (0)	0 (0)	0 (0)	0 (0)	0 (0)	0 (0)	0 (0)	0 (0)	0 (0)	0 (0)	0 (0)
BOS	0 (0)	0 (0)	0 (0)	0 (0)	0 (0)	0 (0)	0 (0)	0 (0)	0 (0)	0 (0)	0 (0)	0 (0)	0 (0)	0 (0)
RAS	0 (0)	0 (0)	0 (0)	0 (0)	0 (0)	0 (0)	0 (0)	0 (0)	0 (0)	0 (0)	0 (0)	0 (0)	0 (0)	0 (0)

Abbreviations: BOS = bronchiolitis obliterans syndrome; LUS = lung ultrasound; L = left LUS zones; RAS = restrictive allograft syndrome; R = right LUS zones.

**Table 3:** Specific HRCT findings according to HRCT areas and stratified corresponding to all, BOS, and RAS patients.

		RU Central	RU Peri-Pheral	RM Central	RM Peri-Pheral	RL Central	RL Peri-Pheral	LU Central	LU Peri-Pheral	LL Central	LL Peri-Pheral
Ground glass opacity	All	9 (36)	12 (48)	8 (32)	11 (44)	8 (32)	10 (40)	9 (36)	10 (40)	9 (36)	8 (32)
	BOS	5 (26)	7 (37)	5 (26)	7 (37)	5 (26)	6 (32)	5 (26)	6 (32)	6 (32)	5 (26)
	RAS	4 (67)	5 (83)	3 (50)	4 (67)	3 (50)	4 (67)	4 (67)	4 (67)	3 (50)	3 (50)
Consolidation	All	6 (24)	7 (28)	5 (20)	8 (32)	4 (16)	7 (28)	7 (28)	9 (36)	4 (16)	6 (24)
	BOS	1 (5)	1 (5)	1 (5)	2 (11)	1 (5)	2 (11)	2 (11)	3 (16)	1 (5)	2 (11)
	RAS	5 (83)	6 (100)	4 (67)	6 (100)	3 (50)	5 (83)	5 (83)	6 (100)	3 (50)	4 (67)
Septal thickening	All	7 (28)	14 (56)	8 (32)	12 (48)	8 (32)	11 (44)	6 (24)	13 (52)	9 (36)	11 (44)
	BOS	2 (11)	8 (42)	3 (16)	7 (37)	3 (16)	5 (26)	2 (11)	8 (42)	3 (16)	5 (26)
	RAS	5 (83)	6 (100)	5 (83)	5 (83)	5 (83)	6 (100)	4 (67)	5 (83)	6 (100)	6 (100)
Reticulation	All	7 (28)	11 (44)	7 (28)	11 (44)	7 (28)	10 (40)	7 (28)	11 (44)	7 (28)	9 (36)
	BOS	1 (5)	5 (26)	1 (5)	5 (26)	1 (5)	4 (21)	1 (5)	5 (26)	1 (5)	3 (16)
	RAS	6 (100)	6 (100)	6 (100)	6 (100)	6 (100)	6 (100)	6 (100)	6 (100)	6 (100)	6 (100)
Noduli	All	1 (4)	1 (4)	0 (0)	1 (4)	0 (0)	1 (4)	0 (0)	1 (4)	0 (0)	0 (0)
	BOS	1 (5)	1 (5)	0 (0)	1 (5)	0 (0)	1 (5)	0 (0)	1 (5)	0 (0)	0 (0)
	RAS	0 (0)	0 (0)	0 (0)	0 (0)	0 (0)	0 (0)	0 (0)	0 (0)	0 (0)	0 (0)
Mosaic attenuation	All	9 (36)	10 (40)	10 (40)	10 (40)	11 (44)	12 (48)	10 (40)	10 (40)	11 (44)	12 (48)
	BOS	8 (42)	9 (47)	9 (47)	9 (47)	10 (53)	11 (58)	9 (47)	9 (47)	10 (53)	11 (58)
	RAS	1 (17)	1 (17)	1 (17)	1 (17)	1 (17)	1 (17)	1 (17)	1 (17)	1 (17)	1 (17)
Tree-in-bud	All	0 (0)	0 (0)	0 (0)	0 (0)	1 (4)	2 (8)	0 (0)	0 (0)	1 (4)	1 (4)
	BOS	0 (0)	0 (0)	0 (0)	0 (0)	1 (5)	2 (11)	0 (0)	0 (0)	1 (5)	1 (5)
	RAS	0 (0)	0 (0)	0 (0)	0 (0)	0 (0)	0 (0)	0 (0)	0 (0)	0 (0)	0 (0)
Bronchiectasis	All	20 (80)	16 (64)	18 (72)	16 (64)	17 (68)	16 (64)	19 (76)	16 (64)	18 (72)	16 (64)
	BOS	14 (74)	12 (63)	13 (68)	12 (63)	12 (63)	12 (63)	13 (68)	12 (63)	13 (68)	12 (63)
	RAS	6 (100)	4 (67)	5 (83)	4 (67)	5 (83)	4 (67)	6 (100)	4 (67)	5 (83)	4 (67)
Peribronchial thickening	All	15 (60)	15 (60)	16 (64)	14 (56)	14 (56)	14 (56)	14 (56)	16 (64)	14 (56)	15 (60)
	BOS	13 (68)	13 (68)	12 (63)	12 (63)	12 (63)	12 (63)	12 (63)	12 (63)	12 (63)	12 (63)
	RAS	2 (33)	2 (33)	4 (67)	2 (33)	2 (33)	2 (33)	2 (33)	4 (67)	2 (33)	3 (50)
Air trapping	All	12 (48)	12 (48)	13 (52)	13 (52)	13 (52)	13 (52)	12 (48)	12 (48)	13 (52)	13 (52)
	BOS	12 (63)	12 (63)	13 (68)	13 (68)	13 (68)	13 (68)	12 (63)	12 (63)	13 (68)	13 (68)
	RAS	0 (0)	0 (0)	0 (0)	0 (0)	0 (0)	0 (0)	0 (0)	0 (0)	0 (0)	0 (0)
Pleural thickening	All	-	11 (44)		11 (44)	-	12 (48)	-	14 (56)	-	11 (44)
	BOS	-	5 (26)		5 (26)	-	6 (32)	-	8 (42)	-	5 (26)
	RAS	-	6 (100)		6 (100)	-	6 (100)	-	6 (100)	-	6 (100)
Honeycombing	All	0 (0)	0 (0)	0 (0)	1 (4)	0 (0)	1 (4)	0 (0)	0 (0)	0 (0)	0 (0)
	BOS	0 (0)	0 (0)	0 (0)	0 (0)	0 (0)	1 (5)	0 (0)	0 (0)	0 (0)	0 (0)
	RAS	0 (0)	0 (0)	0 (0)	1 (17)	0 (0)	0 (0)	0 (0)	0 (0)	0 (0)	0 (0)
Traction bronchiectasis	All	7 (28)	6 (24)	5 (20)	5 (20)	5 (20)	5 (20)	6 (24)	7 (28)	6 (24)	7 (28)
	BOS	1 (5)	1 (5)	0 (0)	1 (5)	0 (0)	1 (5)	0 (0)	2 (11)	1 (5)	2 (11)
	RAS	6 (100)	5 (83)	5 (83)	4 (67)	5 (83)	4 (67)	6 (100)	5 (83)	5 (83)	5 (83)
Volume loss	All	7 (28)	7 (28)	5 (20)	5 (20)	4 (16)	5 (20)	6 (24)	8 (32)	4 (16)	5 (20)
	BOS	1 (5)	1 (5)	1 (5)	1 (5)	1 (5)	1 (5)	1 (5)	2 (11)	1 (5)	1 (5)
	RAS	6 (100)	6 (100)	4 (67)	4 (67)	3 (50)	4 (67)	5 (83)	6 (100)	3 (50)	4 (67)
Fibrosis	All	7 (28)	6 (24)	6 (24)	7 (28)	5 (20)	8 (32)	8 (32)	9 (36)	7 (28)	8 (32)
	BOS	1 (5)	1 (5)	1 (5)	2 (11)	0 (0)	2 (11)	2 (11)	3 (16)	2 (11)	3 (16)
	RAS	6 (100)	5 (83)	5 (83)	5 (83)	5 (83)	6 (100)	6 (100)	6 (100)	5 (83)	5 (83)

Abbreviations: BOS = bronchiolitis obliterans syndrome; LL = left lower area; LU = left upper area; RAS = restrictive allograft syndrome; RL = right lower area; RM = right middle area; RU = right upper area.

**Table 4:** Diagnostic accuracy for IS and pleural thickening according to predefined LUS scanning zones in patients with RAS.

	TP	FP	FN	TN	Sensitivity	Specificity	PPV	NPV
	N	N	N	N	% (95% CI)	% (95% CI)	% (95% CI)	% (95% CI)
**Parenchyma**								
***>=2 zones/lung***								
***IS***	1	0	5	19	17 (0–64)	100 (82–100)	100 (3–100)	79 (58–93)
IS (R/L1 + 4 + 7)	1	0	5	19	17 (0–64)	100 (82–100)	100 (3–100)	79 (58–93)
IS (R/L1 + 7)	0	0	6	19	0 (0–46)	100 (82–100)	-	76 (55–91)
IS (R/L1 + 4)	0	0	6	19	0 (0–46)	100 (82–100)	-	76 (55–91)
IS (R/L4 + 7)	0	0	6	19	0 (0–46)	100 (82–100)	-	76 (55–91)
***>=1 zones/lung***								
IS (R/L1 + 4 + 7)	1	0	5	19	17 (0–64)	100 (82–100)	100 (3–100)	79 (58–93)
IS (R/L1 + 7)	1	0	5	19	17 (0–64)	100 (82–100)	100 (3–100)	79 (58–93)
IS (R/L1 + 4)	1	0	5	19	17 (0–64)	100 (82–100)	100 (3–100)	79 (58–93)
IS (R/L4 + 7)	1	0	5	19	17 (0–64)	100 (82–100)	100 (3–100)	79 (58–93)
**Pleura**								
Thickening (R/L1 + 2 + 3 + 4)	1	0	5	19	17 (0–64)	100 (82–100)	100 (3–100)	79 (58–93)
Thickening (R/L1 + 4 + 7)	2	0	4	19	33 (4–78)	100 (82–100)	100 (16–100)	83 (61–95)
Thickening (R/L1 + 4)	3	0	3	19	50 (12–88)	100 (82–100)	100 (29–100)	86 (65–97)
Thickening (R/L1 + 7)	3	0	3	19	50 (12–88)	100 (82–100)	100 (29–100)	86 (65–97)
Thickening (R/L4 + 7)	2	0	4	19	33 (4–78)	100 (82–100)	100 (16–100)	83 (61–95)
Thickening (R/L1)	4	0	2	19	67 (22–96)	100 (82–100)	100 (40–100)	90 (70–99)
Thickening (R/L4)	3	1	3	18	50 (12–88)	95 (74–100)	75 (19–99)	86 (64–97)
Thickening (R/L7)	5	0	1	19	83 (36–100)	100 (82–100)	100 (48–100)	95 (75–100)
Thickening (R/L1 or R/L7)	6	0	0	19	100 (54–100)	100 (82–100)	100 (54–100)	100 (82–100)
Thickening a.m. IS	4	3	2	16	67 (22–96)	84 (60–97)	57 (18–90)	89 (65–99)

Abbreviations: FN = false negative; FP = false positive; IS = interstitial syndrome; L = left LUS zones; LUS = lung ultrasound; NPV = negative predictive value; PPV = positive predictive value; RAS = restrictive allograft syndrome; R = right LUS zones; TN = true negative; TP = true positive.

## Data Availability

The data presented in this study are available on request from the corresponding author.

## Supplementary Materials

The following are available online at https://www.mdpi.com/2077-0383/10/5/1078/s1, Table S1. Specific pleural LUS findings in RAS and BOS patients; Table S2. Specific parenchymal and pleural HRCT findings in RAS and BOS patients; Table S3. Advantages and disadvantages of HRCT and LUS for subtyping CLAD.
