# Supplementary material for: Lung Ultrasound to Phenotype Chronic Lung Allograft Dysfunction in Lung Transplant Recipients. A Prospective Observational Study
Source: J Clin Med. 2021 Mar 5;10(5):1078. doi: 10.3390/jcm10051078 (PMC7961975; doi:10.3390/jcm10051078)
Supplement: Supplementary file 1 [file jcm-10-01078-s001.pdf]

Table S1. Specific pleural LUS findings in RAS and BOS patients.

Pleura Characteristic	RAS (n = 6)	BOS (n = 19)
Thickening	6 (100.0%)	13 (68.4%)
Fragmented only (any section)	0	1 (5.3%)
Thickening only (any section)	0	4 (21.1%)
Thickening + fragmented (any section)	6 (100.0%)	7 (36.8%)
Average thickness (mm)	5.6 (4 to 9) *	2.9 (1 to 8)

*Wilcoxon rank-sum test: p = 0.0015.

Abbreviations: BOS = bronchiolitis obliterans syndrome; LL = left lower area; LU = left upper area; RAS = restrictive allograft syndrome; RL = right lower area; RM = right middle area; RU = right upper area.

Table S2. Specific parenchymal and pleural HRCT findings in RAS and BOS patients.

Characteristic		RAS	BOS	
		(n = 6)	(n = 19)	p-value (Chi2)
Parenchyma				
Apical fibrosis RUC/RUP/LUC/LUP	N (%)	6 (100)	4 (21)	0.00058
Apical groundglass opacity RUC/RUP/LUC/LUP	N (%)	5 (83)	8 (42)	0.078
Pleura				
Thickening RUC/RUP/LUC/LUP	N (%)	6 (100)	8 (42)	0.013

Abbreviations: BOS = bronchiolitis obliterans syndrome; LUC = left upper central area; LUP = left upper peripheral area; RAS = restrictive allograft syndrome; RL = right lower area; RM = right middle area; RUC = right upper central area; RUP = right upper peripheral area.

Table S3. Advantages and disadvantages of HRCT and LUS for subtyping CLAD.

	Advantages	Disadvantages
**LUS**	Minimal time-consuming	Cannot identify BOS
	Cheap	Only suggestive of PPFE-pattern compatible with RAS
	Easy feasible	
	Frequent monitoring possible	
	Radiation-free	
	High accuracy for pleural pathology	
	May trigger further CLAD examination by identification of the "RAS-sign"	
	Ruling out other pathological findings not related to CLAD	
**HRCT**	Confirmative of radiological RAS and BOS patterns	Radiation risk
	Can quantify pathological pleural and parenchymal affection	Time-consuming
	Ruling out other pathological findings not related to CLAD	Resource heavy (referral and logistics)
		Expensive

Abbreviations: CLAD = chronic lung allograft dysfunction; HRCT = high-resolution computed tomography; LUS = lung ultrasound; PPFE = pleuroparenchymal fibroelastosis; RAS = restrictive allograft syndrome.
